# Employing Explainable AI to Optimize the Return Target Function of a Loan Portfolio

**DOI:** 10.3389/frai.2021.693022

**Published:** 2021-06-15

**Authors:** Thomas Gramespacher, Jan-Alexander Posth

**Affiliations:** Institute for Wealth and Asset Management, School of Management and Law, Zurich University of Applied Sciences, Winterthur, Switzerland

**Keywords:** machine learning, XAI, credit default, P2P lending, FinTech

## Abstract

In the recent years, data science methods have been developed considerably and have consequently found their way into many business processes in banking and finance. One example is the review and approval process of credit applications where they are employed with the aim to reduce rare but costly credit defaults in portfolios of loans. But there are challenges. Since defaults are rare events, it is—even with machine learning (ML) techniques—difficult to improve prediction accuracy and improvements are often marginal. Furthermore, while from an event prediction point of view, a non-default is the same as a default, from an economic point of view much more relevant to the end user it is not due to the high asymmetry in cost. Last, there are regulatory constraints when it comes to the adoption of advanced ML, hence the call for explainable artificial intelligence (XAI) issued by regulatory bodies like FINMA and BaFin. In our study, we will address these challenges. In particular, based on an exemplary use case, we show how ML methods can be adapted to the specific needs of credit assessment and how, in the case of strongly asymmetric costs of wrong forecasts, it makes sense to optimize not for accuracy but for an economic target function. We showcase this for two simple and ad hoc explainable ML algorithms, finding that in the case of credit approval, surprisingly high rejection rates contribute to maximizing profit.

## 1 Introduction

One of the most fundamental properties of risk in finance is its measurability and the fact that one can price it accurately. In the particular case of credit risk, the correct pricing is—for several reasons—quite a challenge: credit and risks as such are asymmetric and skewed toward large losses with low probability and small gains with high probability, resulting in a loss distribution exhibiting the so-called fat tails. Even in traditional banking, these fat tails are quite difficult to assess, and for modeling them correctly, high-quality data and very robust models are needed. This is even truer for the fast-growing peer-to-peer (P2P) lending space where data are quite often sparse, of low quality, and ambiguous (see, e.g., Suryono et al., 2019; Ziegler and Shneor, 2020). At the same time, P2P lenders have a completely different risk profile compared to traditional lenders, namely, although both banks and P2P platforms rely on scoring models for the purpose of estimating the probability of default of a loan, the incentive for model accuracy may differ significantly as in the context of the P2P lending platforms, in most cases, the credit risk is not born by the platform but rather solely by the investors. This, in turn, makes it imperative for the P2P lending platforms to correctly assess their risks, manage them accordingly, and make them transparent to the investors (Ahelegbey et al., 2019b). To overcome the challenges of low-quality, sparse credit data, P2P lenders seek more to employ ML techniques, hoping to achieve by that a higher prediction accuracy of potential defaulters, that is, reduce the already low number of defaults even further (Giudici et al., 2019). As we will detail, this poses a threefold problem: First, optimization in the tail of the loss distribution as such is difficult—because of data issues, error propagation, and accuracy restrictions. Second, just optimizing for the lowest number of defaults conditional to some lower accuracy bound will often result in a suboptimal economic situation, the cost/benefit ratio of defaulters and non-defaulters being highly asymmetrical. Third, the soaring use of advanced ML techniques in finance with the desired goal of higher prediction accuracy renders the decision process increasingly opaque. This, in turn, conflicts with the demands for transparency and explainability issued by regulatory bodies and supervisory authorities in finance (Bafin, 2020). Examining the definition of the “fair” risk premium as the economic payoff function, we immediately understand why:

$$P_{\text{default}\;\text{risk}}^{\text{fair}}=\%-\text{cost}\;\text{of}\;\text{defaults}=\left(1-\text{recovery}\;\text{rate}\right)\times \text{probability}\;\text{of}\;\text{default}.$$ (1)

Here, the cost of comparatively few defaults is balanced by the premium paid by many non-defaulters. This, in turn, implies that omitting a default is very “valuable” and might be done, from an economic point of view, at the cost of forgoing quite a lot of premium-paying business, that is, accepting a high false positive rate (FPR). To better understand this rationale, we only have to look at the most naive estimator for conducting credit business: accept all loans. In this case, the false positive rate will be zero—as will be, unfortunately, the true positive rate (TPR). Since defaults are (relatively) rare events, the accuracy of this naive estimator will be already quite high, although we accepted all of the defaulters. To bring down the number of accepted defaulters, thus increasing the TPR, we have to reject business, but this will go hand-in-hand with an increase of the FPR: since discrimination on a high level of accuracy is increasingly difficult, we will increasingly forgo non-defaulting business and thus income. Consequently, a substantial further gain in prediction accuracy is often simply not achievable, even when employing highly sophisticated ML techniques (compare, e.g., Giudici et al., 2019; Sariev and Germano, 2019; Sariev and Germano, 2020). Already here we can see the fundamental issue: since accepted defaults are much more costly than forgone non-defaulting business, the optimum with regard to the accuracy of predicting the number of defaults never can be the same as the optimum with regard to predicting the highest payoff. While the latter is what matters to any financial institution, the former is what usually gets optimized employing ML algorithms. In this study, we will show that the correct definition of the target function is quite crucial for any credit business and that optimizing along such an economic target function can drastically improve the profitability of the business at hand. Furthermore, we will demonstrate that this concept is quite agnostic with regard to the actual ML technique chosen for optimization and we will discuss this finding in the particular context of explainability and regulatory requirements. Particularly, we will show that the choice of easy-to-understand and intuitively explainable ML algorithms does not substantially compromise prediction accuracy, thus fulfilling both demands: high discriminating and prediction effectiveness as well as explainability. The article will be structured as follows. In Section 2, we will introduce and discuss the dataset we use for our analysis. Section 3 will elaborate on explainable artificial intelligence (XAI), highlight its most prominent characteristics, and comment on its increasing importance. In Section 4, we will employ different ML models to perform the actual prediction and the subsequent optimization, first with regard to number of defaults and second with regard to profit. Here, we will show in detail how different target functions affect the profit and loss (PnL) of the loan portfolio, and we will outline how the different target functions can be formulated for different ML techniques. Last but not least, we will show that this can be done using simple, explainable ML models, without relevant loss of accuracy or fidelity. Section 5 provides a summary and conclusion. Here, we will also outline possible business opportunities for FinTech companies with a clear focus on alternative data.

## 2 Data

**FIGURE 1:**
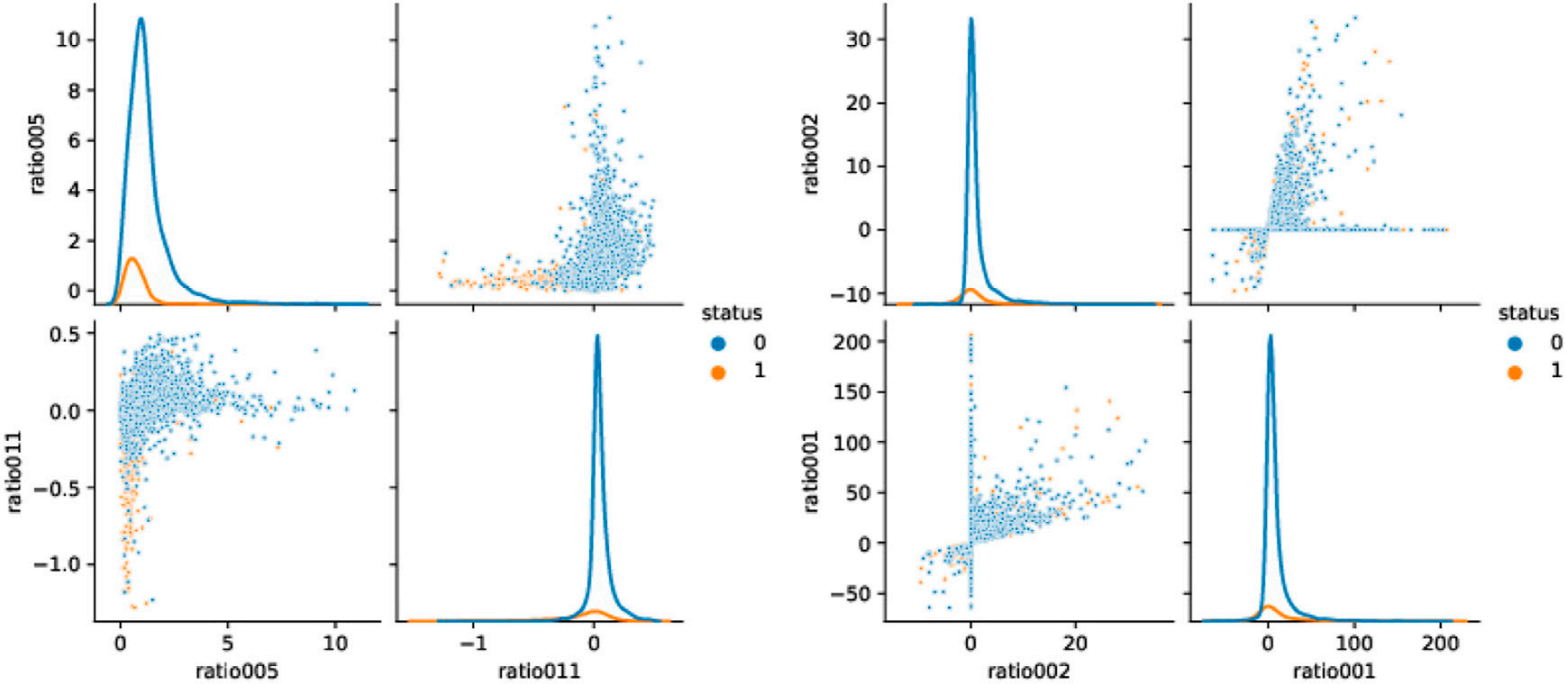
Scatter plots and densities for the two ratios with the best *p*-values and for the two ratios with worst *p*-values, from left to right, respectively.

The data under consideration is the dataset *smaller_dataset.csv* sourced from the FinTech-ho2020 project (www.fintech-ho2020.eu, Fintech-ho2020 (2019-2021)) from the External Credit Assessment Institution (ECAI) (see also Ahelegbey et al., 2019b; Ahelegbey et al., 2019a; Giudici et al., 2019). FinTech-ho2020 is a 2-year project (January 2019–December 2020) that developed a European knowledge-exchange platform aimed at introducing and testing common risk management solutions that automatize compliance of FinTech companies (RegTech) and increase the efficiency of supervisory activities (SupTech). The knowledge exchange platform under the FinTech-ho2020 project consists of SubTech, RegTech, and research workshops. The dataset consists of a total of 4,514 loans, 4,016 or 88.97% of which are not defaulted (indicated by the value 0 of the variable *status* in the dataset), and 498 or 11.03% are defaulted (indicated by the value 1 of the variable *status*). Since the dataset does not contain detailed information about the size of the individual loans, we assume, for simplicity, that all loans are of equal size. If actual individual loan sizes are given, they certainly should be used in the training process of the machine learning algorithm as well as in the calculation of the resulting profit. However, as long as the distribution of loan sizes in the groups of defaulting and non-defaulting loans are roughly identical, replacing the actual loan sizes by their ensemble average seems justifiable. We split the total of 4,514 loans into an (in-sample) training set of 70% (3,159 loans) and an (out-of-sample) test set of 30% (1,355 loans). Each loan is characterized by 24 features of which we use the 19 numerical ones to implicitly infer the creditworthiness of the loans. By this choice, we achieve comparability with and consistency to Giudici et al. (2019). The borrowing information is not raw balance sheet data but ratios of some of those statements. Table 1 lists the 24 loan features of which we use only the 19 highlighted with an asterisk in our further analysis. A first examination of the loan data at hand with respect to the discriminating effectiveness of the 19 features under consideration yields first insights: In Table 2, we have listed the 19 features we will use as discriminating information for the loan decision, sorted ascending by the *p*-values of equal means in the defaulting and non-defaulting subsamples calculated using Welch’s *t* test. As we can see, for example, ratio005 and ratio011 do a pretty good job in differentiating defaults and non-default, while ratio001 and ratio002 do not. This is further corroborated by the density and scatter plots in Figure 1. However, hereinafter, we will always make use of all 19 features and, in the purest sense of the concept of ML, let the algorithm “decide” which feature to use to what extent in its decision “default/non-default.” Nevertheless, we do expect that our machine learning algorithms applied later, especially the highly transparent decision trees, will “learn” to base their predictions mainly on some of the most discriminating features identified here. Utilizing the naive estimator on the loan universe at hand, we immediately can observe the problem inherent in almost all loan data: since defaults are rare events, accepting all loans yields a predictive accuracy of almost 90%; thus, trumping this result using ML techniques will be a challenge from the outset. At the same time, examining the naive case, “accept all loans/all business,” defines our economic base case: Assuming a recovery rate of R=20% and a uniform notional of $1 for all loans for the purpose of our analysis, the cost of defaults in this business case is given by the number of defaulting contracts times the notional times (1−R), that is, $498×(1−20%)=$398.40. Thus, the risk premium necessary to provision for the cost of default can be calculated as follows: $389.40/$4,016=9.92%; here, we assume that the defaulting contracts will also default on their premium payments and hence, the cost of default needs to be entirely paid by the non-defaulting contracts. Assuming further a spread of 500 basis points (bps) is charged on top of the necessary risk premium, an additional income of $4,016×500 bps=$200.80 is achieved. In this naive case, the profit is defined as income generated by payments of the risk premium plus spread minus the cost for defaults. Since here the (expected) loss of default is paid by the risk premium, the profit solely stems from the spread income. This view of the economics of the loan portfolio is akin to the classic credit business of banks.

**TABLE 1:** List of the 24 loan features, with their respective descriptions. Only features marked with * are used as discriminators.

No	ID	Formula
1	ratio001*	(Total assets − shareholder’s funds)/shareholder’s funds
2	ratio002*	(Long term debt + loans)/shareholder’s funds
3	ratio003*	Total assets/total liabilities
4	ratio004*	Current assets/current liabilities
5	ratio005*	(Current assets −current assets: stocks)/current liabilities
6	ratio006*	(Shareholder’s funds + noncurrent liabilities)/fixed assets
7	ratio008*	EBIT/interest paid
8	ratio011*	(Profit (loss) before tax + interest paid)/total assets
9	ratio012*	P/L after tax/shareholder’s funds
10	ratio017*	Operating revenues/total assets
11	ratio018*	Sales/total assets
12	ratio019*	Interest paid/(profit before taxes + interest paid)
13	ratio027*	EBITDA/interest paid
14	ratio029*	EBITDA/operating revenues
15	ratio030*	EBITDA/sales
16	ratio036	Constraint EBIT
17	ratio037	Constraint PL before tax
18	ratio039	Constraint financial PL
19	ratio040	Constraint P/L for period EUR
20	DPO*	Trade payables/operating revenues
21	DSO*	Trade receivables/operating revenues
22	DIO*	Inventories/operating revenues
23	NACE Rev. 2 core code	Industry classification on NACE code, 4 digits precision
24	Turnover*	Revenues

**TABLE 2:** List of the 19 features/ratios used for loan default analysis, sorted ascending by the *p*-values calculated using Welch’s *t* test.

No	ID	Non-default	Default	t-value	*p*-value
1	ratio005	1.243633	0.757088	13.798067	4.900817e-39
2	ratio011	0.047816	−0.133996	14.139802	1.431940e-38
3	ratio012	0.008752	−0.694699	13.210514	9.767516e-35
4	ratio004	1.597079	1.041546	12.834535	2.662133e-34
5	ratio029	0.084345	−0.118976	13.017858	9.449645e-34
6	ratio030	0.091696	−0.119036	12.802153	7.326660e-33
7	ratio003	1.487742	1.086767	11.702571	8.294173e-29
8	ratio008	26.217702	−2.334116	9.623006	1.271021e-20
9	ratio027	40.178063	6.955663	9.116989	7.749628e-19
10	DPO	67.347361	145.180723	−8.947930	6.163742e-18
11	Turnover	3,542.273904	1,749.405622	8.260879	4.223880e-16
12	DSO	91.071215	133.317269	−5.269663	1.955716e-07
13	ratio019	0.211768	6 0.050361	5.145753	3.699050e-07
14	DIO	100.609313	142.471888	−2.003893	4.555395e-02
15	ratio006	7.929163	6.089699	1.423779	1.550459e-01
16	ratio017	1.380640	1.301807	1.412098	1.584418e-01
17	ratio018	1.341287	1.287108	0.948039	3.434978e-01
18	ratio002	1.248618	1.389016	−0.616914	5.375488e-01
19	ratio001	8.852383	9.148855	−0.243575	8.076496e-01

## 3 Discussion on XAI and Econometrics

As widely acknowledged, machine learning (ML) is increasingly used in finance and with very good results at that (Andriosopoulos et al., 2019). One of the prime examples is, of course, the process of credit decisions on sparse datasets of low quality (Lessmann et al., 2015). But there is a fundamental disconnect at the core between machine learning models and statistical or econometric models usually applied when analyzing data (Athey and Imbens, 2019): econometrics is centered around explainability. The very concept starts with an econometric model derived from theoretical reasoning and uses empirical data to “prove” and quantitatively find causal relations between explanatory variables $x_{i}$ and explained variable *y* (Theil, 1971; Greene, 2008). A very simple and hence good example is the linear regression: in a linear regression, the significance, the sign, and the size of the parameters matter. The value of the parameters should represent true partial effects of single inputs. In a model that explains defaults of loans given the features of the loan like notional, loan-to-value (LTV), and delay-in-payment, an increase in the delay-in-payment should lead, ceteris paribus, to a higher probability of default. For a model to be able to achieve this explanatory power, a lot of model assumptions have to be fulfilled and thoroughly tested. Especially, a theoretical, economic model is needed that is rigorously analyzed with regard to its model diagnostics like variable selection, functional form, analysis of residuals, and many more. In machine learning, on the other hand, accuracy in prediction is the primary goal (Athey, 2018). Here, explanations, partial effects, and causal relations are usually not of central interest. In the case of image recognition, for example, there is no theoretical model (and no need for such) that would come up with the causal relation and functional dependence of individual pixels colors (the $x_{i}$) to the output variable (the *y*) determining if there is a cat in the picture or not. Instead, ML tries to fit sufficiently flexible and complex models, often with thousands or tens or hundreds of thousands of parameters called the hypothesis space, to the data in a way to optimize predictive accuracy. As long as the result is sufficiently accurate, the user is usually only too happy to accept the ML technique as a “black box.” Indeed, it can be argued that the big advantage of ML is exactly its capability to infer correct and useful but non-explainable results from a not-ordered profusion of input data, to “see something,” some relation in the data that is not necessarily accessible by theory and hence, not obviously apparent to the human mind. Furthermore, demanding some level of explainability from the ML techniques employed might compromise their proficiency and result in a suboptimal outcome. The challenge here then is to retain the effectiveness of ML while at the same time providing some insights into its inner workings. The level of abstraction at which the ML techniques operate and produce results heavily depends on the context—as does the need for explainability. It does not originate within the concept of machine learning per se. In many circumstances, and especially in finance, users of ML models would not or could not simply accept the output of a black box model, but would require at least some level of interpretability or explainability of the model before it could be used (Ribeiro et al., 2016; Carvalho et al., 2019). A good example here, again, is the credit or loan business where decisions have to be comprehensible and explainable to customers, regulators, and, last but not least, to the banks’ own management alike (Bussmann et al., 2020). The customer might want to know why his application for a loan was rejected and might even have a legal right to demand this information, the regulator needs to make sure that this information is made available by the loan originator, and the loan originator might want to better understand their underwriting process or simply make sure that they comply with regulations. From the regulators’ and the banks’ point of view also, risk management considerations play an important role: ML models have been known in the past to learn their decision-making based on “wrong” information which led to the “correct” result purely because of coincidence or because having been applied to a biased dataset (see, e.g., the “husky” vs. “wolf” example in Ribeiro et al., 2016). In this case, the opaqueness of the ML model implies a model error of unknown severity – an operational risk which no bank or loan originator should feel comfortable with. The regulator, on the other hand, will be concerned with the systemic component of this risk and force the risk takers not only to provide transparency but also to understand their own risks to the maximum extent possible. Finally, it has been proven that purely ML-based decision processes—by automatic evaluation of the data at hand—sometimes “learn” to discriminate by characteristic that simply are not considered socially tolerable, like, for example, by race or gender. To overcome and/or prevent the abovementioned problems, some degree of transparency and explainability of the ML algorithm is required. This is where explainable artificial intelligence (XAI) comes into play: It either highlights the inherent transparency of a ML model or supplies a tool-set of methods that, applied to a (complex) fully trained ML model, adds some level of explanation to the outputs of the model (e.g., by providing an input–output sensitivity analysis) (see Carvalho et al., 2019; Arrieta et al., 2020). In XAI, it is distinguished between different levels of exlainability (Arrieta et al., 2020): the most basic level is to understand the functional relationship between inputs and outputs. This, in principle, is possible because for every trained ML model, there is a fixed, deterministic mathematical or functional relation between inputs and outputs. However, in very complex models like neural nets or random forests, this relation may be way too complicated to be directly understood by the human mind. In this case, one is reduced to conduct an implicit analysis, that is, one is not able to understand the ML algorithm’s decision process in detail, but can only examine how outputs change with regard to specific, predefined changes of the input set. Such a sensitivity analysis then may at least provide some insights into the dynamics of the model and validate high-level rationales (see, e.g., Arrieta et al., 2020; Ribeiro et al., 2016; Lundberg and Lee, 2017). For “mathematically simple” models (like a linear or logistic regression), on the other hand, the functional dependence is sufficiently simple to be directly understood by humans. In the same way, the prediction process of a single, not too deeply branched decision tree is still directly comprehensible. Here, we can understand each detail, each step of the decision process of the ML model and validate it accordingly. Furthermore, should in such a model the need arise, we can restrict it to such characteristics that are not socially discriminatory. In this study, we illustrate how ML can be used to optimize a bank’s economic target function while accepting or rejecting credit business, and still be explainable. For simplicity, we use two ML models that are *ad hoc* explainable: a logistic regression model and a single decision tree model. Thus, both objectives are met: full and straightforward explainability from the outset as well as optimization of an economic target function employing ML.

## 4 Optimizing With Machine Learning

In this section, we will use two machine learning models, a logistic regression and a decision tree, to identify defaulting and non-defaulting loan contracts. We keep both models as simple as possible: first, because this guarantees a certain level of ad hoc explainability as discussed in the previous section, and second, because more elaborate models often fail to significantly improve the models’ performance while quickly compromising its transparency. For the same reason, we do not try to optimize the models by hyperparameter tuning. We use a standard logistic regression model without regularization^1^ and a simple decision tree of depth 3 grown without any further constraints.^2^ For both models, we use all of the ratios included in the dataset as features (c.f. Section 2) and the variable *status* as the label to be predicted. We use 70% of the data to train the models and 30% as a test set to evaluate the models’ out-of-sample performance.^3^

### 4.1 Logistic Regression

We use the following logistic regression model to predict a contract’s probability of default given its features $x_{1},\ldots ,x_{n}$:

$$p\left(x_{1},\ldots ,x_{n}\right)=\frac{1}{1-\exp\left(\beta_{0}+\sum_{i=1}^{n}\beta_{i}x_{i}\right)}.$$ (2)

**FIGURE 2:**
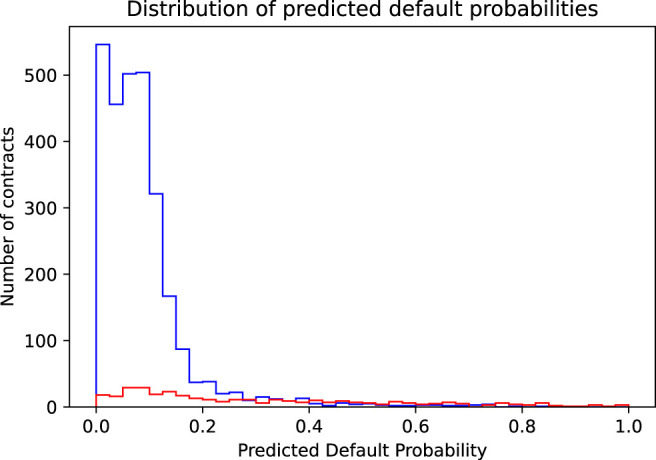
Shown is the distribution of the predicted default probabilities separately for contracts that actually do not default (blue histogram) and for contracts that actually do default (red histogram).

The parameters $\beta_{0},\beta_{1},\ldots ,\beta_{n}$ are learned by fitting the model to the training set, that is, by minimizing the standard cross-entropy cost-function of the logistic regression. Figure 2 shows the distribution of the predicted default probabilities in the training set for contracts that actually did default (red histogram) and did not default (blue histogram). We immediately observe the small number of actually defaulting contracts compared to the many non-defaulting ones. For most of the non-defaulting contracts, the model reasonably predicts small default probabilities below 20%. However, rather small default probabilities are also (wrongly) predicted for quite some of the contracts that actually did default.^4^ To finally arrive at a classification, that is, to be able to decide which contracts should be accepted as business and which should be rejected, we have to introduce a threshold $p_{\text{thr}}$ on the predicted default probability. Contracts with predicted default probabilities below the threshold are accepted and those above the threshold are rejected. A threshold $p_{\text{thr}}=0$ would reject all the contracts. That would be the correct decision for all the actually defaulting contracts but the wrong decision for all the non-defaulting ones, thus leading to a prediction accuracy identical to the (low) ratio of defaulting contracts in the sample (approximately 0.11). On the other hand, a threshold $p_{\text{thr}}=1.0$ would accept all contracts, that is, would be identical to the naive model discussed in Section 2, leading to a prediction accuracy identical to the (high) ratio of non-defaulting contracts in the sample (approximately 0.89).

#### 4.1.1 Accuracy Maximizing Threshold

**FIGURE 3:**
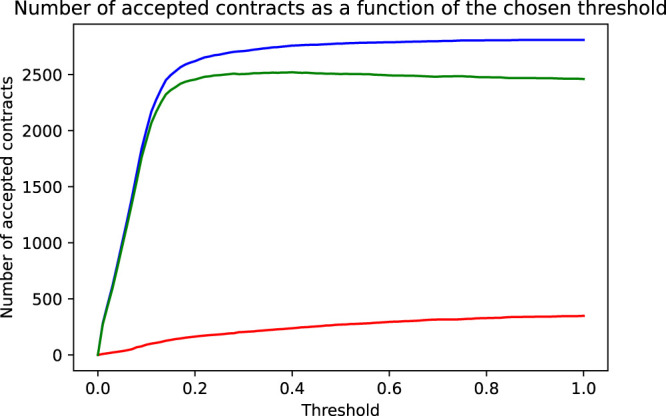
As a function of the chosen threshold are shown the number of correctly accepted non-defaulting contracts (blue line), the number of incorrectly accepted defaulting contracts (red line) and the net effect, that is, the difference of correct and incorrect decisions reflecting the models accuracy (green line).

We now proceed to find the threshold that leads to the highest possible prediction accuracy. Mathematically speaking, this simply means to maximize the target function “prediction accuracy” as a function of the chosen threshold $p_{\text{thr}}$. The accuracy is $\text{Accuracy}=1/N\sum_{i=1}^{N}{\text{Accuracy}}_{i}$ with the indicator function.

$${\text{Accuracy}}_{i}=\{\begin{array}{ccc} 1 & \text{if} & {\hat{y}}_{i}=y_{i}\\ 0 & \text{if} & {\hat{y}}_{i}\neq y_{i} \end{array},$$ (3)

where ${\hat{y}}_{i}$ is the threshold dependent predicted class of loan *i* and $y_{i}$ is its true status. We graphically illustrate this process using Figures 2, 3: As we increase the threshold above 0, we start to correctly accept more and more non-defaulting contracts (corresponding to the area under the blue histogram curve in the range along the *x*-axis from 0 to the selected threshold in Figure 2). This increases the prediction accuracy. On the other hand, we also start to wrongly accept more and more of the defaulting contracts (corresponding to the area below the red histogram), which reduces the accuracy. The optimal, accuracy-maximizing threshold corresponds exactly to the point, where the area below the red histogram starts to increase faster than the area below the blue histogram, that is, when a further increase of the threshold would accept more defaulting than non-defaulting contracts. In our sample, this point is reached at a threshold of $p_{\text{thr}}=0.4$. Figure 3 shows in more detail how the model’s accuracy changes as the threshold is varied from 0 to 1: It shows the number of accepted contracts and the resulting effect on the prediction accuracy as a function of the chosen threshold. The steep rise in the blue curve shows the many non-defaulting contracts that are correctly accepted when the threshold is increased above 0. The red curve shows the number of wrongly accepted defaulting contracts. The green curve finally shows the net effect, the difference of correct (blue) and wrong (red) decisions, thus representing the behavior of the model’s prediction accuracy. It reaches its maximum, that is, achieves maximum accuracy, at a threshold of 0.4. However, as seen in this figure, the maximum is not very pronounced. In fact, any threshold in a range between maybe 0.15 and 0.6, and, in particular, also the often chosen default threshold of 0.5, would lead to a very similar accuracy. Table 3 provides a summary of the performance of the logistic regression model at different thresholds. The first column shows the in- and out-of-sample performance of the naive model accepting all contracts (corresponding to a logistic regression model with a threshold $p_{\text{thr}}=1.0$). The second column shows the performance of a model using the accuracy maximizing threshold $p_{\text{thr}}=0.4$, while the naive model accepts all contracts; the accuracy maximizing model can identify and reject roughly one third of the defaulting contracts (111 true positives). Unfortunately, it also wrongly rejects nearly 2% of the good, non-defaulting contracts (51 false positives). In the prediction accuracy, these two effects nearly cancel out each other (relative to the naive case only 60 more contracts, that is, 1.9% of all contracts, are correctly classified) so that the accuracy of the accuracy maximizing model is only increased by 1.9 percentage points relative to the naive model. Therefore, taking only into consideration the slightly improved accuracy, it would appear pretty questionable to the bank’s management if employing a (potentially costly to implement and maintain) machine learning model in the credit approval process would make sense. However, since the economic (dollar) costs of wrong decision are very different for wrongly accepted defaulting and wrongly rejected non-defaulting contracts, we see a significant increase in the profit of the accuracy maximizing model vs. the naive model. Identifying and rejecting at least some of the defaulting contracts has an enormous positive impact on the bank’s profitability that, by far, outweighs the loss incurred by rejecting some of the non-defaulting contracts. Even though rejecting good business does not seem attractive, the significant increase in the dollar amount of the bank’s profit is a strong argument for employing a machine learning model. An important observation is that all of the above observations, including the significant increase in profit, are not only seen in the training set (in-sample) but also in the test set (out-of-sample)—which has not been used, neither in the training of the model nor in optimizing the threshold.

**TABLE 3:** Overview of the in- and out-of-sample performance figures of logistic regression models with different thresholds $p_{\text{thr}.}$

		naive	Accuracy maximizing	Profit maximizing
		$p_{\text{thr}}=1.0$	$p_{\text{thr}}=0.40$	$p_{\text{thr}}=0.17$
In-sample	# Contracts	3,159	3,159	3,159
	# Accepted contracts	3,159	2,997	2,713
	Acceptance rate	1	0.9487	0.8588
	# True positives	0	111	202
	True-positive rate (TPR)	0	0.3181	0.5788
	# False positives	0	51	244
	False-positive rate (FPR)	0	0.0181	0.0868
	Accuracy	0.8895	0.9085	0.8762
	Profit	140.06	221.25	265.26
	Δ profit relative to naive		58.0%	89.4%
Out-of-sample	# Contracts	1,355	1,355	1,355
	# Accepted contracts	1,355	1,278	1,153
	Acceptance rate	1	0.9432	0.8509
	# True positives	0	52	84
	True-positive rate (TPR)	0	0.3490	0.5638
	# False positives	0	25	118
	False-positive rate (FPR)	0	0.0207	0.0978
	Accuracy	0.8900	0.9100	0.8649
	Profit	60.74	98.61	110.33
	Δ profit relative to naive		62.3%	81.6%

#### 4.1.2 Weighting of Contracts and Profit Maximization

We have seen above that using an accuracy maximizing threshold improves the model’s accuracy only slightly but did increase the resulting profit considerably. This was mainly due to the fact that the model was able to greatly reduce the bank’s loss by correctly identifying and rejecting defaulting contracts. This gives rise to the idea to try to choose the threshold not in a way to maximize accuracy, but in a way to directly maximize profit. Mathematically speaking, this means maximizing the profit target function $\text{Profit}=\sum_{i=1}^{N}{\text{Profit}}_{\text{i}}$, where

$${\text{Profit}}_{i}=\{\begin{array}{ccc} 0.1492 & \text{if} & {\hat{y}}_{i}=0\;\text{and}\;y_{i}=0\\ -0.8 & \text{if} & {\hat{y}}_{i}=0\;\text{and}\;y_{i}=1\\ 0 & \text{if} & {\hat{y}}_{i}=1 \end{array}.$$ (4)

**FIGURE 4:**
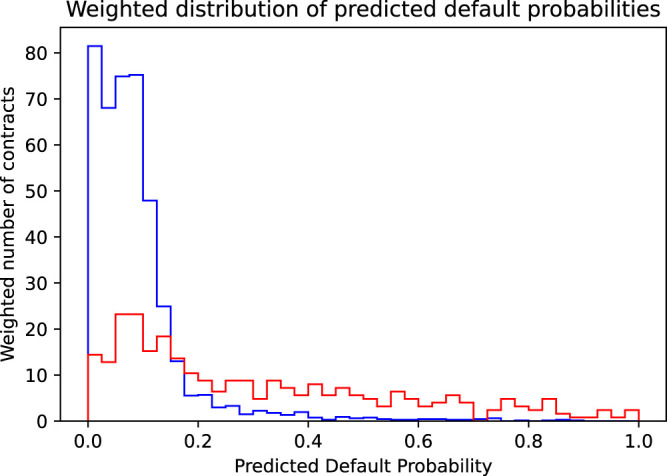
Shown is the distribution of the predicted default probabilities, where the distribution for the actually non-defaulting contracts (blue histogram) is weighted by their income (corresponding to the risk premium plus the spread) and the distribution of the actually defaulting contracts (red histogram) is weighted by their loss (corresponding to the face value reduced by the recovery rate).

**FIGURE 5:**
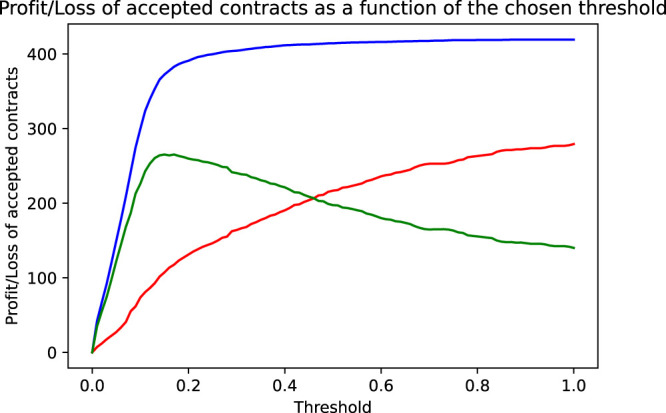
As a function of the chosen threshold are shown the income by the accepted non-defaulting contracts (blue line), the loss incurred by the accepted defaulting contracts (red line), and the net effect, that is, the bank’s profit (income less loss, green line).

Again, we illustrate this maximization process graphically. The starting point to find the accuracy maximizing threshold was Figure 2, showing the distribution of the predicted default probabilities. Implicitly and by construction, in this histogram, every contract is given the same weight. This is the appropriate view if we want to judge the model’s accuracy where every wrong or correct decision contributes equally. However, if we focus on profit, we must consider the completely different cost of wrongly accepted defaulting contracts vs. the cost of wrongly denied non-defaulting contracts. The solution is to weigh each contract with the dollar cost resulting if it is wrongly classified, that is, we weigh the non-defaulting contracts by the income we lose, if we reject them ($0.149 income from risk premium plus spread), and the defaulting contracts by the loss we incur if we accept them ($0.80, equal to the face value of the contract reduced by the recovery rate of 20%). Figure 4 shows the resulting histogram. We immediately see the relative increase of the importance of the defaulting contracts (red histogram) compared to the equally weighted distribution in Figure 2. Using the same reasoning as in the section above, we also see immediately that the optimal threshold, that is, the crossover point of the histograms of the defaulting and non-defaulting contracts, now is at a much lower value. We find an optimal threshold $p_{\text{thr}}=0.17$. Pushing the threshold higher than this value, the loss of wrongly accepted additional defaulting contracts outweighs the income generated by additionally accepted good business. Figure 5 shows the profit or loss of accepted contracts as the threshold is increased above 0. The blue curve shows the profit generated by earning the risk premium plus spread on accepted non-defaulting contracts. The red curve shows the loss incurred by accepted but defaulting contracts. The green curve finally shows the net effect, that is, the profit of the non-defaulting contracts less the loss generated by the defaulting ones. At a threshold of 1 (accepting all contracts), we get the profit of the naive model $140.06. At a threshold of 0 (rejecting all contracts), we have no business at all and thus also zero profit. However, in between these two extremes, at the threshold $p_{\text{thr}}=0.17$, a very pronounced maximum is reached with a profit of $265.26. We also see that while the profit achieved using the accuracy maximizing threshold 0.40 is larger than the profit of the naive case, it can be considerably further increased by using the profit maximizing threshold of 0.17. Staying with the often used threshold of 0.5 would, however, yield a profit quite a bit smaller than the one achieved by the accuracy maximizing model and considerably smaller than the one achieved by the profit maximizing model. The third column in Table 3 summarizes the performance figures of the profit maximizing model. It has a quite low acceptance rate (roughly 15% of all contracts are rejected). However, it manages to correctly identify and reject more than half of the defaulting contracts. This comes at the cost of wrongly rejecting many of the good, non-defaulting contracts (nearly 10% of the good business is rejected). Consequently, the model’s accuracy is (slightly) reduced compared to the accuracy maximizing model. However, the model’s profit could be increased considerably since it was able to identify many more of the defaulting contracts. Even though, at first sight, using a threshold as low as 0.17 appears to be quite unattractive since too much of the bank’s potential business is rejected, a second look on the bank’s profit proves the opposite: even with this lower business volume, the bank’s profit (in absolute dollars) is substantially increased. It is again important to note that the above conclusions also hold true out-of-sample, that is, in a setting that corresponds to the model judging completely new, as yet unseen credit applications.

#### 4.1.3 Visualization of Accuracy and Profit Maximization in the ROC Diagram

**FIGURE 6:**
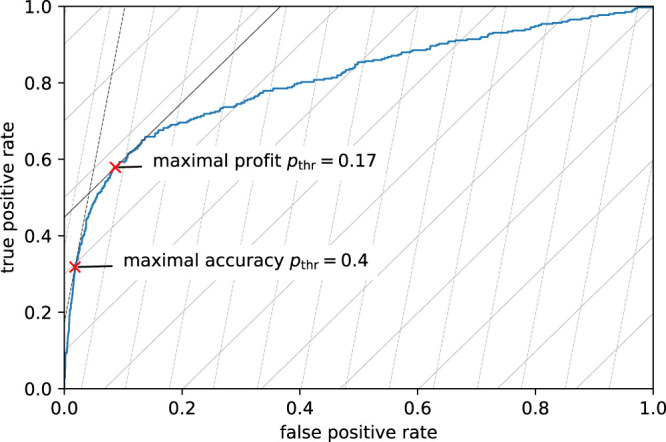
In-sample ROC curve of the logistic regression model (blue curve). Added are the curves of equal accuracy (steep dashed lines) and the curves of equal profit (flat solid lines). The lines more to the upper left represent spots of higher accuracy and profit, lines more to the lower right represent spots of lower accuracy and profit. The two red crosses represent the TPR and FPR of models with thresholds that lead to maximal accuracy or maximal profit.

The logistic regression model predicts probabilities and allows for a continuous shift of the decision threshold. Such a model’s quality is often judged by looking at its receiver operating characteristic (ROC). Figure 6 shows the in-sample ROC diagram of our logistic regression model. The ROC curve shows the model’s true positive rate (TPR) and false positive rate (FPR) for thresholds between 0 and 1. This curve starts in the lower left corner at a threshold of 1.0, representing a model that accepts all contracts and consequently has 0 TPR (no defaulting contracts are identified) and 0 FPR (no non-defaulting contracts are wrongly rejected). As the threshold is lowered below 1.0, more and more contracts are rejected. This leads to a (fast) increase of the TPR (many defaulting contracts start to be correctly identified) but also to a (slow) increase of the FPR (some non-defaulting contracts are wrongly rejected). Together, this leads to a steep increase of the ROC curve. As the threshold is decreased further, the increase of the TPR slows down, while the increase of the FPR speeds up, leading to a flattening of the ROC curve until it reaches a TPR and FPR of 1 at the threshold 0 (rejecting all contracts). The quality of the logistic regression model can be judged based on the shape of the ROC curve. The more the ROC curve is bent to the upper left corner (maximal TPR with minimal FPR), the better the model. The performance figure is the area under the ROC curve, the AUC. For our logistic regression model, the in-sample AUC is 0.8058. However, the choice of the best possible threshold depends on the target function to be optimized. Both of the target functions we investigate, accuracy and profit maximization, depend on the number of correct and wrong decisions made by the model, that is, on the model’s TPR and FPR. The steep dashed lines in Figure 6 represent combinations of TPR and FPR that yield the same accuracy. We call these iso-accuracy lines. Since in our training sample there are approximately 8 times more non-defaulting contracts than defaulting contracts, an increase of the FPR by one percentage point must be compensated by an increase of the TPR by about 8 percentage points to achieve the same accuracy. Thus, the iso-accuracy lines have a slope of approximately 8. Iso-accuracy lines close to the upper left corner of the diagram correspond to high accuracy, while those that are closer to the lower right corner correspond to lower accuracies. The model’s best possible threshold is, therefore, achieved at the TPR/FPR combination where the iso-accuracy line lays tangent to the model’s ROC curve (c.f. dark dashed line in Figure 6). This point is indicated in Figure 6 by a red cross and corresponds to a threshold of 0.4 where the model achieves a TPR of 0.3181 and an FPR of 0.0181 (c.f. Table 3). As the main focus of the bank is not to maximize prediction accuracy but rather to maximize its profit, we added in the ROC diagram lines of equal profit, iso-profit lines (flat solid lines in Figure 6). Since wrongly accepted defaulting contracts are about five times as expensive as wrongly rejected non-defaulting contracts, the slope of the iso-profit lines is by a factor of 5.36 lower than the slope of the iso-accuracy lines. The slope of the iso-profit lines is 8/5.36≈1.5. The optimal, profit maximizing threshold is again found at the point where the iso-profit line is tangent to the model’s ROC curve (c.f. dark solid line in Figure 6). This point is more to the right along the ROC curve and reached at the much lower threshold of 0.17 with a TPR of 0.5788 and an FPR of 0.0868.

### 4.2 Predicting Defaults Using a Single Decision Tree

As an alternative to the logistic regression model, we illustrate the steps necessary to calibrate to a profit maximizing model using another machine learning model, a single decision tree. Even though this model is even simpler than the logistic regression and, thus, also more explainable and transparent in its decisions than the logistic regression model, it is still capable to yield significant performance improvements by tuning it to profit maximization. We use a simple tree with a depth of 3 and minimize the Gini impurity measure to grow the tree. No further restrictions were imposed while growing the tree.

#### 4.2.1 Tuning the Tree Growth to Maximize Accuracy and Profit

**FIGURE 7:**
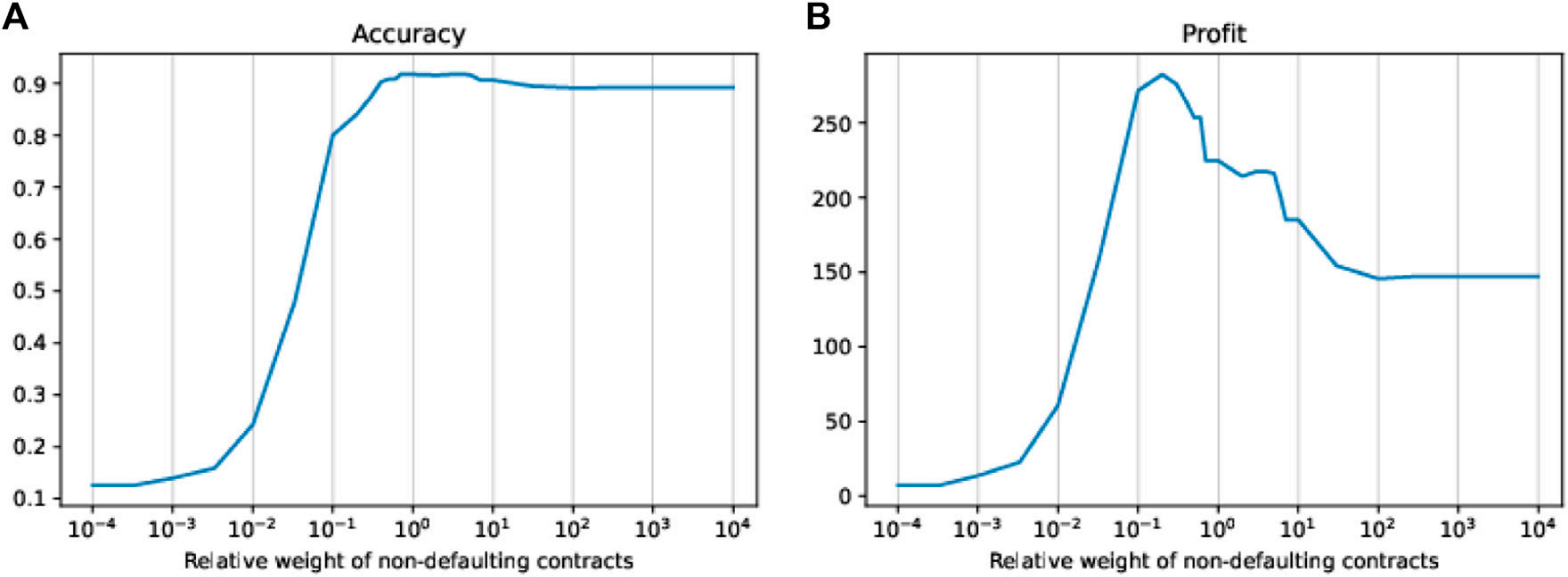
In-sample accuracy **(A)** and in-sample profit **(B)** of trees as a function of different relative weights of the non-defaulting contracts.

Since the tree we use is quite shallow (depth of 3 leading to just eight leaves), it does not make a lot of sense to tune the decision threshold applied in the leaves to decide whether to accept or reject a contract. We, therefore, leave the threshold fixed at 0.5, that is, whether a new contract is accepted or rejected is decided upon the simple majority of the contracts of the training set in the leaf to which the new contract is assigned. However, we can tune the growth of the decision tree by using weights for the two classes of contracts in the training data. We do this by tuning the relative weight of the non-defaulting contracts relative to the defaulting ones in a range from $10^{-4}$ to $10^{4}$. The left-hand side of Figure 7 shows the resulting in-sample accuracy of trees as a function of the relative weight applied to the non-defaulting contracts. A very low weight, that is, more or less neglecting non-defaulting contracts altogether, leads to a tree that basically rejects all contracts (corresponding to a logistic regression model with a threshold close to 0), whereas a high weight, that is, more or less neglecting the defaulting contracts, leads to a tree that accepts nearly all contracts (corresponding to the naive model or a logistic regression model with a threshold close to 1). While for these extreme weights the accuracy approaches the ratio of defaulting contracts (for a weight of $w=10^{-4}$ the accuracy is approximately 0.11) or non-defaulting contracts (for a weight of $w=10^{4}$ the accuracy is approximately 0.89), it has a (shallow) maximum at a weight close to 1. In fact, the maximum accuracy is reached for weights between 0.7 and 4, where the maximum in-sample accuracy of 0.9174 is reached. The second column of Table 4 shows the in-sample and out-of-sample performance of the decision tree grown using the accuracy maximizing weight of 1 (i.e., using the original, unaltered training dataset). As with the logistic regression model, compared to the naive model, we see only a slight improvement in accuracy but a considerable improvement in the other performance figures achieved by correctly identifying and rejecting about one-third of the defaulting contracts. Note also that compared to the accuracy maximizing logistic regression model, the simple decision tree shows, in some figures, even a slightly better performance. In particular, the tree has a similar true positive rate as the logistic regression model, but a much lower false positive rate. Due to the asymmetric costs of wrong classification of defaulting and non-defaulting contracts, it seems natural to assume that a corresponding over-weighting of the defaulting contracts (or under-weighting of non-defaulting contracts) should grow a tree whose decisions lead to higher profits. As accepting a defaulting contract is more than 5 times as costly than rejecting a non-defaulting contract, a relative weight of the non-defaulting contracts of approximately 1/5=0.2 seems reasonable to assume. The right-hand side of Figure 7 shows the in-sample profit achieved by trees grown using different relative weights *w* of the non-defaulting contracts. We clearly see a sharp maximum at a relative weight somewhat above 0.1. A closer inspection of the numbers shows that, as expected, the maximum profit of $282.54 is actually achieved at a weight of w=0.2.^5^ The third column of Table 4 shows the in- and out-of-sample performance of the tree grown with a dataset using a relative weight of 0.2 for the non-defaulting contracts. It is especially remarkable that in-sample, the profit maximizing tree achieves a higher dollar value of profit than the profit maximizing logistic regression model, even though the tree rejects more than 20% of the contracts. However, the out-of-sample profit (which is the relevant quantity for the estimation of the bank’s future profit) of the logistic regression and the tree are very similar.

**TABLE 4:** Overview of the in- and out-of-sample performance figures of decision tree models with different weightings *w* of the non-defaulting contracts.

		naive	Accuracy maximizing	Profit maximizing
		w=∞	w=1.0	w=0.2
In-sample	# Contracts	3,159	3,159	3,159
	# Accepted contracts	3,159	3,027	2,498
	Acceptance rate	1	0.9582	0.7908
	# True positives	0	110	254
	True-positive rate (TPR)	0	0.3152	0.7278
	# False positives	0	22	407
	False-positive rate (FPR)	0	0.0078	0.1448
	Accuracy	0.8895	0.9174	0.8411
	Profit	140.06	224.78	282.54
	Δ profit relative to naive		60.5%	101.7%
Out-of-sample	# Contracts	1,355	1,355	1,355
	# Accepted contracts	1,355	1,296	1,088
	Acceptance rate	1	0.9565	0.8030
	# True positives	0	44	91
	True-positive rate (TPR)	0	0.2953	0.6107
	# False positives	0	15	176
	False-positive rate (FPR)	0	0.0124	0.1459
	Accuracy	0.8900	0.9114	0.8273
	Profit	60.74	93.7	107.28
	Δ profit relative to naive		54.3%	76.6%

#### 4.2.2 Comparison of the Accuracy Maximizing and Profit Maximizing Trees

**FIGURE 8:**
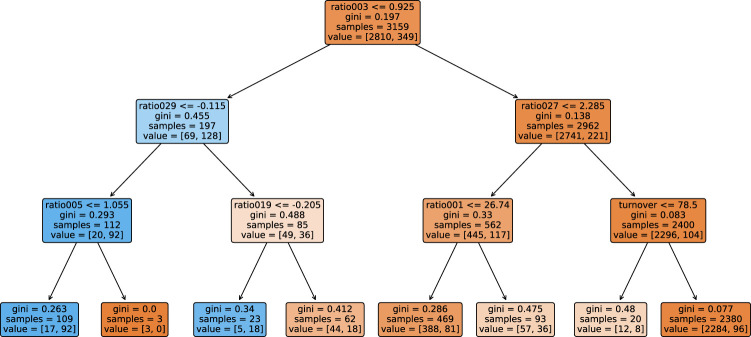
Tree grown using the dataset, where non-defaulting and defaulting contracts are weighted equally. This tree maximizes prediction accuracy.

**FIGURE 9:**
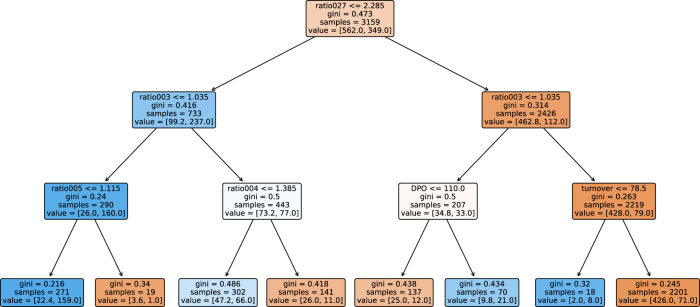
Tree grown using the dataset, where the weight of the non-defaulting contracts is reduced by a factor of 0.2 relative to the defaulting contracts. This tree maximizes the banks profit.

Figures 8, 9 show the decision trees at the accuracy maximizing weight of 1 and the profit maximizing weight of 0.2. The accuracy maximizing tree identifies approximately one third of the defaulting contracts only in leaves where defaults represent a clear majority. The remaining two thirds of defaults remain hidden in leaves where they are mixed with a majority of non-defaulting contracts. Even though a large majority of the defaulting contracts is wrongly classified due to the low number of defaults in our highly unbalanced dataset, this still results in a tree with maximum in- and out-of-sample prediction accuracy. However, for the task of profit maximization, the defaults are assigned a much larger (relative) weight. Therefore, the profit maximizing tree “tries much harder” to correctly identify and separate more of the defaulting contracts. Using only four of the available 19 ratios, namely *ratio003*, *ratio029*, *ratio005*, and *ratio019*, the accuracy maximizing quickly arrives at branches and leaves with clear majorities indicated by the more saturated colors in Figure 8. The profit maximizing tree needs six different ratios, namely *ratio027*, *ratio003*, *ratio005*, *ratio004*, *DPO*, and *turnover*, and still ends up in quite some leaves with rather weak majorities indicated by the less saturated colors. It is instructive to note that, confirming our expectation, most of the ratios used in the branches of the trees are among the most significant discriminators as identified in our statistical analysis in Table 2. In fact, the most relevant ratios as identified during the growth process of the tree are asset-liability ratios (ratio003 and ratio005) and earnings ratios (ratio027 and ratio029). This is consistent with the traditional wisdom of banking experts that these types of ratios are among the most relevant ones when it comes to judging the credit worthiness of a company.

## 5 Summary and Conclusion

For many applications, as for example, credit decisions made by banks, the output of the used models has to be sufficiently transparent and understandable. This often prevents or at least complicates the use of many advanced and nontransparent machine learning models. However, especially in the case of highly unbalanced datasets one is typically confronted with in credit applications, already the naive procedure (simply basing the decision upon the majority class in the training data) or the use of simple, transparent and ad hoc explainable machine learning algorithms can easily achieve high prediction accuracy that is difficult to significantly be enhanced by more complex and nontransparent algorithms. On the other hand, for users of machine learning models, it is often not prediction accuracy which is of most concern, but each application has its own, business specific target function to be optimized. In the use-case studied in this study, the relevant target function is the profit the bank can draw from its credit business. While application of simple machine learning algorithms only minimally improves prediction accuracy over the naive case of accepting all business, it quickly shows a considerable positive effect on the banks profit by identifying and rejecting some of the defaulting contracts. However, neither the often applied pure accuracy maximization nor the balancing of the training dataset to equal shares of defaulting and non-defaulting contracts leads to the maximal profit. In order to maximize profit, it is crucial to include the user’s target function in the choice of the best possible model and parameters. In the case of the logistic regression, we tuned the threshold distinguishing between accepted and rejected contracts to maximize the given profit target function. In case of the decision tree, we used weighting to balance the data not to equal shares of both types of contracts but to reflect the impact of the model’s correct and wrong decisions on the target function, the bank’s profit. As a result, we have seen that applying simple machine learning algorithms that are tuned to profit maximization can increase the banks profit on new business considerably. For both models studied, the bank’s profit could be increased by up to 80% relative to the naive case of accepting all business. We observe that the profit-maximizing models tend to reject surprisingly many of the contracts, that is, these models accept a lot of falsely rejected good business in order to sort out a few more of the defaulting contracts. This is because the cost of a wrongly accepted defaulting contract by far outweighs the loss incurred by falsely rejected good, non-defaulting contract (forgone business). With our dataset and models, up to 20% of all contracts are rejected, approximately two thirds of which are actually non-defaulting contracts. This could lead to new business opportunities, for example, for peer-to-peer lending platforms, that might try to use additional alternative data and advanced machine learning techniques to identify some of the remaining good business within the many contracts rejected by banks. To conclude, from a purely theoretical point of view, the observations made in this study of course can be translated to any use case where one has to deal with unbalanced datasets and target functions that depend in a highly asymmetrical way on the model’s decisions.

## Data Availability

Publicly available datasets were analyzed in this study. These data can be found here: www.fintech-ho2020.eu, Fintech-ho2020 (2019–2021), smaller_dataset.csv, https://github.com/danpele/FINTECH_HO_2020/tree/main/3.%20Use%20Cases/1.%20Big%20Data%20Analytics/Use%20Case%20I_BDA/Replication_code_BDA_I.

## Footnotes

1 Optimal L2 regularization using cross-validation did not significantly change the performance of the model (measured in terms of AUC), nor would it change any of our discussions and conclusions made below.

2 Using cross-validation, we found the optimal depth to be in a range between two and four splits. Larger trees tend to overfit the training data while smaller trees significantly underperform.

3 We use a stratified split to keep the ratio of defaulting contracts in the training set and test set identical.

4 In fact, 93% of the non-defaulting contracts had predicted default probabilities below 20%, while this was the case for only 47% of the defaulting contracts.

5 Note that balancing the training data to equal shares of defaulting and non-defaulting contracts would correspond to a weight of w=0.1 and to a profit of $272.75.
